# Modulation of Glutathione Hemostasis by Inhibition of 12/15-Lipoxygenase Prevents ROS-Mediated Cell Death after Hepatic Ischemia and Reperfusion

**DOI:** 10.1155/2017/8325754

**Published:** 2017-07-24

**Authors:** Moritz Drefs, Michael N. Thomas, Markus Guba, Martin K. Angele, Jens Werner, Marcus Conrad, Christian J. Steib, Lesca M. Holdt, Joachim Andrassy, Andrej Khandoga, Markus Rentsch

**Affiliations:** ^1^Department of General, Visceral, Vascular and Transplant Surgery, University Hospital Munich, Ludwig-Maximilians University of Munich, Campus Großhadern, Munich, Germany; ^2^Institute of Developmental Genetics, Helmholtz Zentrum München, German Research Center for Environmental Health, Munich, Germany; ^3^Department of Internal Medicine II, University Hospital Munich, Ludwig-Maximilians University of Munich, Campus Großhadern, Munich, Germany; ^4^Institute of Laboratory Medicine, University Hospital Munich, Ludwig-Maximilians University of Munich, Campus Großhadern, Munich, Germany

## Abstract

**Background:**

Reactive oxygen species- (ROS-) mediated ischemia-reperfusion injury (IRI) detrimentally impacts liver transplantation and resection. 12/15-Lipoxygenase (12/15-LOX), an antagonistic protein of the glutathione peroxidase 4 (GPX4) signaling cascade, was proven to mediate cell death in postischemic cerebral and myocardial tissue. The aim of this study was to investigate the impact of 12/15-LOX inhibition on hepatic IRI.

**Methods:**

Livers of C57BL/6 mice were exposed to 60 minutes of partial warm ischemia and 90 minutes of reperfusion after previous Baicalein administration, an inhibitor of 12/15-LOX. Tissue samples were analyzed by TUNEL assay, Western blot, and spectral photometry.

**Results:**

TUNEL labeling showed a significant reduction of hepatic cell death following baicalein pretreatment. Western Blot analysis revealed a significant downregulation of Jun-amino-terminal-kinase (JNK), caspase-3, and poly-ADP-ribose-polymerase (PARP), besides considerably lowered p44/42-MAP-kinase (ERK1/2) expression after Baicalein administration. A significant elevation of glutathione oxidation was measured in Baicalein pretreated livers.

**Conclusion:**

Our data show that inhibition of 12/15-lipoxygenase causes significant cell death reduction after hepatic ischemia and reperfusion by enhancing glutathione metabolism. We conclude that GPX4-dependent cell death signaling cascade might play a major role in development of hepatic IRI, in which the investigated proteins JNK, caspase-3, ERK1/2, and PARP might contribute to tissue damage.

## 1. Introduction

Hepatic ischemia-reperfusion injury (IRI) is associated with significant morbidity and mortality and occurs during liver resection, transplantation, and trauma, as well as septic and hemorrhagic shock [1–3]. IRI is characterized by a complex cascade of intracellular signaling processes leading to different forms of cell death mainly due to reoxygenation during reperfusion (oxidative stress) [4, 5]. These include spontaneous and programmed cell death with or without initiating an inflammatory response, defined by the morphological or biochemical behavior of the cell and may be commonly classified as apoptosis, necrosis, necroptosis, and ferroptosis. Partial overlapping and the simultaneous occurrence of the different forms of cell death are known to be possible [6–8]. In recent years, newly detected nonapoptotic forms of cell death, most notably ferroptosis, became more and more important in understanding the complex characteristics of cell death [9–11]. By all means, the accumulation of reactive oxygen species (ROS) inducing oxidative stress represents a strong inducer for many forms of cell death [9, 12–14]. This ROS-mediated liver damage not only leads to limited postoperative liver function but is also regarded as an immediate origin of allograft rejection, delayed graft function, and primary nonfunction after liver transplantation [2, 15]. Therefore, several antioxidant treatments are currently under investigation to improve the outcome after liver transplantation [16].

Among the proposed mechanisms of oxidative stress, uncontrolled lipid peroxidation may represent a link between intracellular reactive oxygen species and membrane disruption [17–19], which is by a major part mediated by mitochondrial permeability transition [8, 20]. 12/15-Lipoxygenase (12/15-LOX) has been considered to be a relevant source of lipid peroxidation in IRI models, as it deoxygenates membrane-bound arachidonic acid—most importantly in mitochondrial membranes [21]—into 12S/15S-HpETE (12S/15S-hydroperoxyeicosatetraenoic acid) [22] which is one of the key metabolites to induce cell death after induction of IRI [23]. Glutathione peroxidase 4 (GPX4) may be regarded as cell protective and functionally antagonistic to 12/15-LOX in IRI, as it is the only GPX to process phospholipid hydroperoxides in membranes [24], thus being able to reduce 12S/15S-HpETE into 12S/15S-HETE (12S/15S-hydroxyeicosatetraenoic acid) which will not trigger further cell death [23]. These findings were supported by cell-based experiments showing that treating GPX4^−/−^ cells with HpETE led to significantly accelerated loss of cell viability whereas the addition of HETE had no impact on cell death [25]. Furthermore, it has been shown that GPX4 activity itself can be inhibited by sustained oxidative stress as intracellular stores of reduced glutathione are oxidized resulting in GPX4 inhibition, increased lipid peroxidation, and cell death [26]. This mechanism has been reconfirmed in vivo in several models after neuronal [27–29] and myocardial ischemia and reperfusion [30]. In a murine experimental model, mice overexpressing GPX4 are protected against oxidative stress [31], and administration of glutathione, the main substrate of GPX4, attenuates IRI after warm ischemia [32, 33]. These results were furthermore confirmed by Shang et al. providing evidence in 2016 that impaired glutathione biosynthesis was associated with a higher level of oxidative stress and hence with a higher impact of IRI on liver damage [34]. Analysis of the downstream pathway using selective knockout mice showed that GPX4 controls lipid peroxidation presumably via regulation of 12/15-lipoxygenase activity which in turn was shown to contribute to cell death [26].

Lipid peroxidation during oxidative stress in hepatic IRI has been attributed to the activity of 5-lipoxygenase [35]. Therefore, 5-lipoxygenase inhibitors have been investigated in detail throughout recent years and have been shown to reduce the postischemic leukotriene-mediated hepatic injury, mainly by reduction of leukotriene B4 [36–38]. However, in contrast, little is known about the role of the 12/15-lipoxygenase signaling system and its downstream mediators on cell death following hepatic ischemia and reperfusion, although inhibition of 12/15 lipoxygenase is feasible by administration of the antioxidant Baicalein, a flavonoid which is contained in the Japanese herbal supplement Sho-saiko-to and whose interventional potential as an antioxidant has been investigated for several years [39–41].

Therefore, the present study aimed at investigating the impact of the 12/15 lipoxygenase inhibition with Baicalein on cell death induction after experimental hepatic ischemia and reperfusion in the mouse.

## 2. Material and Methods

### 2.1. Experimental Model and Technique

For all experiments, 6- to 8-week-old male C57BL/6 wild-type mice (Charles River, Sulzfeld, Germany) were used. All experiments were carried out according to the German legislation on protection of animals under the Protocol number 55.2.1.54-2532-100-11. The surgical procedure and experimental protocol were performed in dependence on the techniques described by Hori et al. in 2011 [42].

Briefly, under commonly used anaesthesia with midazolam, medetomidine, and fentanyle, a polypropylene catheter was inserted into the right carotid artery in a retrograde direction for measurement of mean arterial pressure and heart rate, as well as for volume substitution with normal saline. A warm (37°C) reversible ischemia of the right anterior, left anterior, and left posterior liver segment was induced for 60 min by clamping the common supplying pedicle using a microclip beginning 10 min after laparotomy, resulting in an ischemic fraction of 65% of the liver [42].

Reperfusion time was 90 min in all experiments. Three IR groups and three corresponding sham-operated groups were analyzed (*n* = 10 each).

Baicalein, the inhibitor of 12/15-LOX (Merck, Germany), was administered intraperitoneally 30 minutes before laparotomy, in order to allow a well-established duration for adequate peritoneal drug distribution and absorption [43, 44]. The dosage was 120 mg/kg body weight (group 1), equaling an injected volume of 150 *μ*l at maximum solubility in dimethylsulfoxide (DMSO) according to manufacturer's instructions. To avoid objectionable interactions caused by rise of intra-abdominal pressure, the intraperitoneally administered volume did not exceed the limits recommended for laboratory animal care [45, 46]. Controls were treated either with a corresponding volume of the vehicle (DMSO; AppliChem, Germany) (group 2) or left untreated, receiving a corresponding volume of saline intraperitoneally (group 3).

Blood samples were taken from the vena cava at the end of the experiment and immediately centrifuged at 2000 ×g for 10 minutes. The obtained serum was immediately stored at −80°C. Tissue specimens from I/R and non-I/R liver lobes were collected at 90 minutes after reperfusion for histological investigations and Western blot analyses.

### 2.2. Serum Analyses

Serum bilirubin, creatinine, cholinesterase, glutamate dehydrogenase (GLDH), aspartate aminotransferase (AST), and alanine aminotransferase (ALT) activities were determined at 37°C with an automated analyzer (Hitachi 917, Roche-Boehringer, Germany) using standardized test systems (ALAT-FS, ASAT-FS, Bilirubin Auto Total-FS, Creatinine-FS, GLDH-FS-DGKC, DiaSys, Germany).

### 2.3. TUNEL Assay

Cells undergoing cell death were quantified in formalin-fixed and paraffin-embedded tissue slices of the hepatic left posterior segment, harvested as described above. TUNEL staining was performed using an “In Situ Cell Death Detection Kit, POD” (Roche, Switzerland) according to the manufacturer's instructions. Counterstaining to visualize DNA fragmentation was performed using the 4′,6-diamidino-2-phenylindole technique (DAPI, Vector Laboratories Inc., USA). Quantification of TUNEL-positive cells was performed by means of planimetric analyses of labeled cells with fluorescence microscopy using an emission wavelength of 450–500 nm and fluorescence detection at 515–565 nm (green light). 10 randomly selected regions of interest (ROI) of each tissue slice were analyzed separately under 10x enlargements. The microscopic images gained by this manner were consecutively processed using ImageJ (National Institutes of Health, USA), thus allowing the software to automatically detect and calculate the area fraction of TUNEL-positive cells for each group as a direct proportional parameter for dead cell count. All histological examinations were performed by one of the authors in a blinded manner.

### 2.4. Western Blot

Western blots were used for detection of proapoptotic protein levels in the liver lobes undergoing ischemia reperfusion injury. Tissue of the hepatic right anterior segment was minced in buffer containing 1 μl/ml dithiothreitol (DTT, Sigma-Aldrich, USA) and 10 μl/ml protease inhibitor mixture containing aprotinin (100 U/ml, Sigma-Aldrich, USA). Protein concentration in each hepatic tissue lysate was determined using a “Pierce™ BCA Protein Assay Kit” (ThermoFisher Scientific, USA). SDS-gel electrophoresis and protein transfer onto nitrocellulose membranes and followed by overnight incubation with antibodies at 4°C were performed for activity detection of stress-activated protein kinase/Jun-amino-terminal kinase (SAPK/JNK, rabbit, 46/54 kDa, Cell Signaling Technology, USA), mitogen-activated protein kinase p44/42 (ERK1/2, rabbit, 42/44 kDa, Cell Signaling Technology, USA), caspase-3 (rabbit, 17/19/35 kDa, Cell Signaling Technology, USA), and poly-ADP-ribose-polymerase (PARP, rabbit, 89/116 kDa, Cell Signaling Technology, USA). ERK1/2 and SAPK/JNK antibodies were analyzed by blotting their respective phosphorylated and total fractions; all Western blots were blotted a second time using a glyceraldehyde 3-phosphate dehydrogenase (GAPDH) antibody (rabbit, 37 kDa, Cell Signaling Technology, USA) in order to achieve a reliable loading control. All Western blots were processed using an HRP-linked antibody (anti-rabbit IgG, Cell Signaling technology, USA) as secondary antibody. Blotted results were visualized by chemiluminescence technique (SuperSignal, ThermoFisher Scientific, USA).

### 2.5. Measurement of GSH and GSSG

Intracellular concentrations of glutathione (GSH) and its oxidized state glutathione disulfide (GSSG) were analyzed applying spectral photometry and a kinetic test first described by Tietze in 1969 [47]. Tietze's test was performed with tissue of shock frozen liver lobes that underwent IRI and were lysed by adding perchloric acid and ethylenediaminetetraacetic acid (EDTA). Protein precipitate was consecutively removed from the centrifuged tissue homogenate. In the following procedure, the total glutathione concentration was determined by measuring the velocity of thionitrobenzoic acid (TNB) accumulation with spectral photometry at 412 nm representing the product of a spontaneous reaction between GSH within the tissue sample (hepatic right anterior segment) and 5,5-dithiobisnitrobenzoic acid (DTNB), which was added to the standardized reaction. This velocity is directly proportional to the total glutathione concentration (GSH + GSSG) in the sample. GSSG concentration could be determined using the same testing mechanism as described above, yet after preventing autooxidation of GSH to GSSG by adding N-ethylmaleimide (NEM). NEM conjugates GSH potently as first described by Tietze and furtherly developed by Lauterburg et al. in 1984 [48]. Accordingly, intracellular concentration of GSH can be calculated by subtracting measured GSSG levels from total glutathione levels. Using this technique, both substrate and product of GSH metabolizing selenoperoxidases can be estimated indirectly, among which GPX4 plays a distinct role as suggested by others and described above.

### 2.6. Statistical Analysis

Statistics and ANOVA were calculated using Brown-Forsythe test, Bartlett's test, Fisher's LSD-test, and Dunnett's & Tukey's multiple comparisons test (GraphPad Prism 7, GraphPad Software, San Diego, USA). Mean values ± SEM are given. *P* values less than 0.05 were considered significant.

## 3. Results

### 3.1. Effects of Baicalein and DMSO Pretreatment on Macrohemodynamics

**Figure 1:**
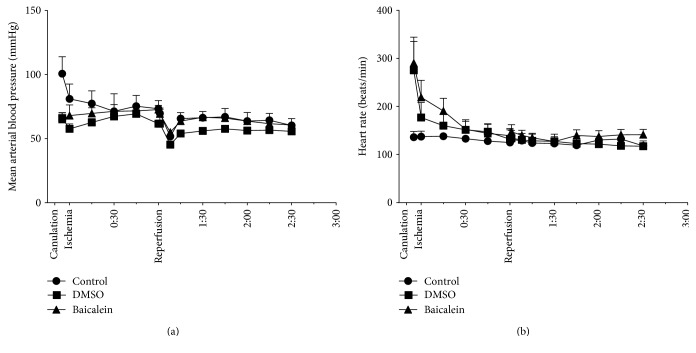
Measurements of macrohemodynamics while inducing ischemia and reperfusion in the liver throughout the experiment. Intra-arterial measurements of blood pressure (a) and heart rate (b) revealed a brief decrease in mean arterial blood pressure by 35.4% at the beginning of the experiment after Baicalein administration and after sole DMSO pretreatment (−34.4%) compared to the control. A heart rate elevation at the same time point by 104.2% (Baicalein) and 93.7% (DMSO) compared to control could also be measured. In further progress, no significant differences in macrohemodynamics between the groups could be monitored. A self-limiting decrease of blood pressure five minutes after reperfusion could repeatedly be observed in all groups. Data are presented as the mean ± SEM.

Assessment of macrohemodynamics has been performed throughout the entire experiment via intra-arterial measuring of blood pressure and heart rate. Obvious differences between the experimental groups could not be observed throughout the majority of the duration of the experiment. Solely at the beginning of the experiment, directly after cannulation of the carotid artery and thereby immediately after drug administration, a significant change in hemodynamics could be monitored for a considerably short period of time (Figure 1). At this time point, after sole DMSO pretreatment, the blood pressure was by −35.4% significantly lower (*p* < 0.001) and the heart rate was significantly higher (+93.7%; *p* < 0.001) compared to the control group. No significant alteration was recorded after additional Baicalein application. Within the following five minutes, both blood pressure and heart rate aligned among the different groups, so that no significant differences could be monitored. A dip of blood pressure five minutes after reperfusion of the liver was observed in each experimental group repeatedly, representing a loss of systemic blood volume into the reperfusing liver lobes. This proved to be a quickly self-adjusting process as the decrease of blood pressure resolved within approx. two minutes (Figure 1).

### 3.2. Effects of Baicalein and DMSO Pretreatment on Cell Death after Hepatic Ischemia and Reperfusion

**Figure 2:**
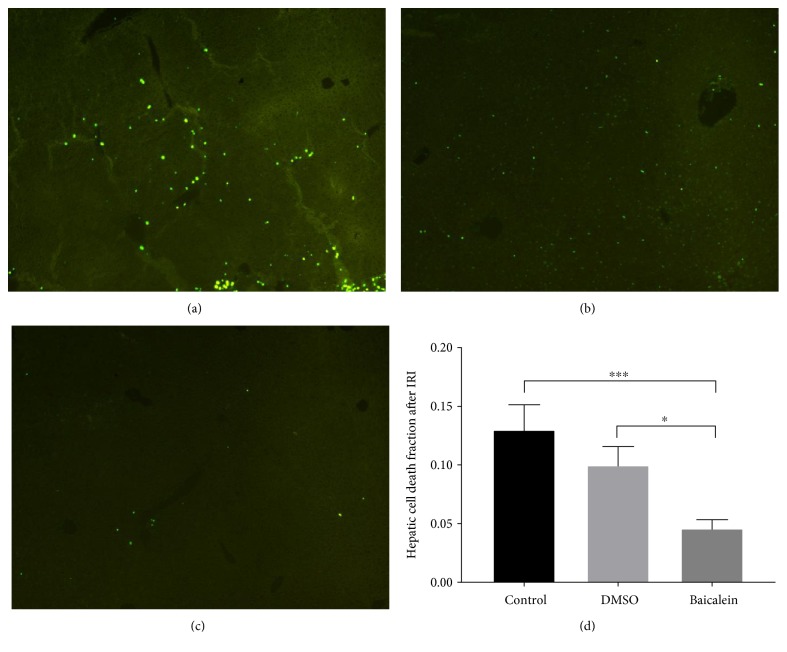
TUNEL assay of liver tissue (left posterior segment) exposed to ischemia-reperfusion injury. Representative images of TUNEL stainings demonstrating the differences in cell death after no/saline pretreatment (a), pretreatment with DMSO (b), and pretreatment with Baicalein (c). Quantitive planimetric analyses of TUNEL positive cells confirm a significant reduction of cell death after Baicalein pretreatment (Baicalein versus control by −64.8%, ^∗∗∗^*p* = 0.0007; Baicalein versus vehicle DMSO by −54.1%, ^∗^*p* = 0.0471) (d). Data are presented as the mean ± SEM.

Analyses of hepatic tissue samples processed with TUNEL assay after inducing an ischemia reperfusion injury as described above revealed relevant differences in loss of cell viability between the groups (Figure 2). Baicalein pretreatment resulted in a significant decrease in area fraction of the TUNEL-positive signals within the liver tissue by −64.8% in contrast to the control group (*p* = 0.0007). Interestingly, a slight decrease in cell death could also be demonstrated after the sole application of the vehicle for Baicalein, DMSO by −23.2%. This result, however, did not reach statistical significance. Even though taking the slight decrease of cell death induced by the sole application of DMSO into account, the prevention of cell death by Baicalein remained evident, as TUNEL-positive cell count in the Baicalein group was significantly lowered by −54.1% in contrast to the DMSO group (*p* = 0.0471). Therefore, these data suggest that cell death after hepatic ischemia and reperfusion is significantly lowered by Baicalein administration.

### 3.3. Effects of Baicalein and DMSO Pretreatment on Proapoptotic Enzyme Pathways

**Figure 3:**
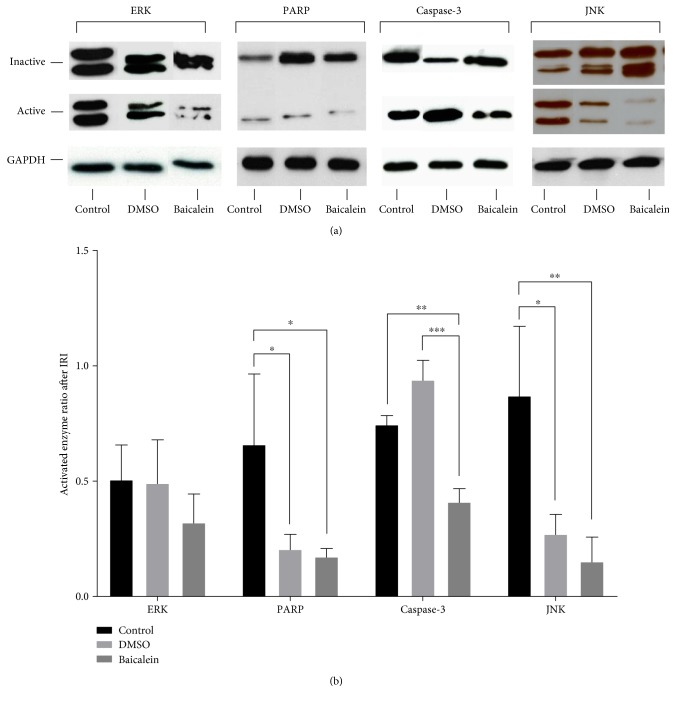
Western blot assays of liver tissue (right anterior segment) exposed to ischemia-reperfusion injury for detection of activity levels of proapoptotic proteins JNK (stress-activated protein kinase/Jun-amino-terminal kinase), ERK (mitogen-activated protein kinase p44/42), caspase-3, and PARP (poly-ADP-ribose-polymerase). (a) Representative images of Western blots demonstrating the differences in enzyme activity levels for each experimental group. Inactive proteins are shown on top for ERK (total ERK, 42 + 44 kDa), PARP (uncleaved, 116 kDa), caspase-3 (loading control GAPDH, 37 kDa), and JNK (total JNK, 46 + 54 kDa). Activated protein forms are shown in the middle for ERK (phosphorylated ERK, 42 + 44 kDa), PARP (cleaved, 89 kDa), caspase-3 (cleaved, 20 kDa), and JNK (phosphorylated JNK, 46 + 54 kDa). Loading controls are shown below in each group for glyceraldehyde 3-phosphate dehydrogenase (GAPDH, 37 kDa). (b) Quantitative photometric analyses of Western blots prove the mostly significant downregulation of proapoptotic enzyme activity levels after Baicalein pretreatment. Activated enzyme ratio is calculated as a quotient of active/inactive protein form. Changes in protein level activation after sole vehicle administration (DMSO) could also be observed. Data are presented as the mean ± SEM. ^∗^*p* < 0.05; ^∗∗^*p* < 0.01; ^∗∗∗^*p* < 0.001.

Potential molecular pathways leading to differences in cell death responses as mentioned above have been analyzed by measuring several proapoptotic enzyme levels within the liver tissue that underwent ischemia and reperfusion. These measurements indeed revealed distinctive differences in enzyme activity levels between the different experimental groups (Figure 3).

Mitogen-activated protein kinase p44/42 (ERK) activity was evaluated as a ratio of activated phosphorylated ERK over the total measurable ERK amount. A discrete negative regulatory trend by −36.7%, though statistically not significantly, could be monitored in the activation of ERK after Baicalein pretreatment, whereas the vehicle DMSO showed no influence.

Administration of Baicalein also resulted in a −73.8% significantly lower activity of PARP (poly-ADP-ribose-polymerase), measured as a quotient of cleaved—thus activated—PARP over uncleaved PARP, compared to the control group (*p* = 0.0307). Concurrently, a significant downregulation of PARP activity by −68.8% after sole DMSO application could be monitored (*p* = 0.0435).

In contrast, caspase-3 activation was found to be slightly upregulated after DMSO application, albeit statistically not significantly. Baicalein pretreatment, however, led to a highly significant decrease in caspase-3 activity by −45.0% compared to the control group (*p* = 0.004). Hence, our data suggest that the antiapoptotic effect of Baicalein by downregulation of caspase-3 can be estimated to be even higher due to the primary elevation of caspase-3 activity by its vehicle DMSO. Comparing the results of the Baicalein group to those of the DMSO group expectedly showed an even more distinct and significant decrease in the level of active caspase-3 (−56.4%, *p* = <0.0001).

The ratio of phosphorylated JNK (Jun-amino-terminal kinase) versus total JNK expression in liver tissue correlates with JNK activity. After Baicalein pretreatment, a decrease of JNK activity could be evaluated by −82.6%, representing a highly significant result (*p* = 0.0049). Also, the sole vehicle administration of DMSO resulted in a significantly lowered JNK activity, yet not as significantly as in combination with Baicalein (−68.9%, *p* = 0.0222).

In summary, Baicalein affected several different pro-cell death pathways after hepatic IRI in an inhibiting manner.

### 3.4. Effects of Baicalein and DMSO Pretreatment on Liver Damage Parameters in Blood

**Figure 4:**
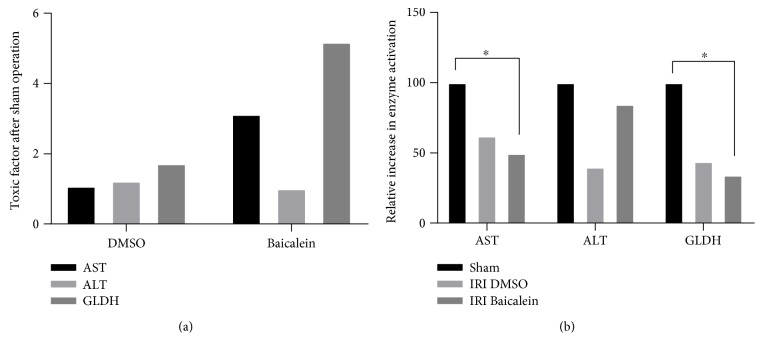
Quantitative serum analyses of caval blood gained after inducing hepatic IRI or, respectively, after sham operation. (a) Quantification of the increase of liver enzymes in sham-operated mice after DMSO and Baicalein administration by calculating the toxic factor. The toxic factor is calculated as a ratio of enzyme elevation after drug administration compared to the control group over the control group itself. The data show that Baicalein and DMSO administration relevantly increase AST, ALT, and GLDH levels. (b) Assessment of attenuation of the liver enzyme elevation post DMSO and Baicalein pretreatment after inducing hepatic IRI by calculating a relative increase in enzyme activation. This is generated as ratio of the calculated toxic factor after IRI over the previously calculated toxic factor of the respective sham-operated mice. Therefore, the relative enzyme increase for the sham group matches 100% for each enzyme and pretreatment to be investigated. Hence, it can be stated that Baicalein pretreatment after IRI leads to an acknowledgeable decrease of liver enzyme elevation compared to the respective sham-operated mice, significant for AST activation (−50.3%; ^∗^*p* = 0.0438) and GLDH activation (−65.8%; ^∗^*p* = 0.0481).

Potential toxicity of administered drugs and systemic liver response was investigated by evaluating serum levels of several liver enzymes, biliary excretion, and hepatic synthesis parameters, as well as renal function. No significant differences in serum levels of creatinine, bilirubin, or cholinesterase could be found between the groups, neither after sham operation nor after inducing IRI (Table 1). However, after application of Baicalein and DMSO solely, a distinct increase in serum levels of AST, ALT, and GLDH could be observed both in sham-operated mice and in those after hepatic ischemia and reperfusion. For further evaluation, the factor by which an administered agent elevates the amount of liver enzyme release in pretreated and subsequently sham-operated mice compared to the sham-operated control group without pretreatment was calculated. This has been performed for each of the liver enzymes mentioned above and described as the particular toxic factor (Figure 4). Therefore, it could be shown that AST levels were elevated by 3.13-fold, ALT levels by 1.01-fold, and GLDH levels by 5.18-fold after Baicalein pretreatment. DMSO administration showed an increase in enzyme levels by a factor of 1.08 for AST, 1.22 for ALT, and 1.72 for GLDH. After inducing hepatic IRI, a relevant increase of liver enzymes could also be measured in each group (Table 1). Further, it has been evaluated whether the toxic factor described above could be attenuated by a potential hepatoprotective effect of the administered drugs after inducing IRI by calculating a relative liver enzyme decrease. Therefore, a ratio of the calculated toxic factor after IRI over the previously evaluated toxic factor of the respective sham group as described above was generated. Again, this has been performed for each pretreatment drug and each liver enzyme to be scrutinized. Thus, the toxic factor of an administered drug for one specific enzyme in the sham group represents a relative toxic factor of 100% for the respective evaluation after inducing IRI. Using this method, a significant attenuation of liver enzyme increase following Baicalein pretreatment by −50.3% for AST (*p* = 0.0438) and −65.8% for GLDH (*p* = 0.0481) as well as a negative regulatory trend of −15.4% for ALT could be observed after hepatic IRI. DMSO administration led to a −38.0% decrease of elevated AST, −60.0% of ALT, and −56.0% of GLDH release. Therefore, it can be stated that DMSO and Baicalein pretreatment cause an elevated release of liver enzymes AST, ALT, and GLDH after both sham operation and hepatic IRI, whereas the increase is clearly attenuated after IRI induction.

### 3.5. Effects of Baicalein and DMSO Pretreatment on Glutathione Hemostasis

**Figure 5:**
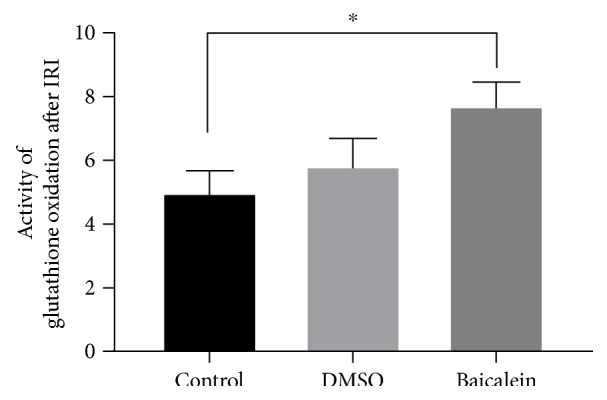
Quantification of spectral photometric analyses of intracellular activity of glutathione oxidation using Tietze's kinetic test. Activity was calculated as a ratio of measured amount of oxidized glutathione disulfide (GSSG), the product of GPX4, over its substrate glutathione (GSH). Pretreatment with Baicalein showed a significant increase in glutathione oxidation by +54.5% (^∗^*p* = 0.0438), DMSO solely in a more discrete fashion (+16.7%).

The direct influence of the administered agents Baicalein and DMSO on glutathione-dependent peroxidases was evaluated by measuring its substrate GSH and its product GSSG and subsequently calculating a quotient of GSSG over GSH as an indirect measure of glutathione peroxidase activity. The pretreatment with Baicalein resulted in a significant intrahepatic elevation of glutathione oxidation by 54.5% (*p* = 0.0438) (Figure 5). Relevant differences in activation could be monitored between Baicalein and its vehicle, as the sole application of DMSO led to an increase of glutathione oxidation by not more than 16.7%. Even though this result could not be proven to be statistically significant, a positive regulatory trend of DMSO on glutathione peroxidase activity could be speculated. However, a significant direct impact of Baicalein pretreatment towards upregulation of oxidative glutathione consumption could be monitored.

## 4. Discussion

Hepatic IRI is one of the most common causes for organ dysfunction and failure after orthotopic liver transplantation (OLT) and may compromise the outcome after extended liver resections and OLT [15, 16, 49]. Therefore, strategies to minimize the negative effects of ischemia are now in the forefront of clinical and experimental studies. After the attenuation of the effects of cold ischemia in OLT has been a crucial approach in transplant research for a long period, warm ischemia has attracted considerable interest throughout recent years as it is known to affect predominantly parenchymal liver cells and biliary injury [50–52]. Different modes of cell death are known to play a role in hepatic IRI [8, 20]. For a successful therapy of hepatic IRI, it is important to know which mode of cell death takes place and which pathophysiological mechanism underlies this damage. Apoptosis is still being considered as one of the main forms of cell death after hepatic IRI although several different studies now show that there is substantial overlap with other forms of regulated necrotic cell death, for example, due to partially overlapping mechanisms such as mitochondrial membrane transitions [20, 53]. Regulated forms of necrosis in hepatic IRI are widely regarded to elicit an inflammatory response unlike apoptosis. Due to the rupture of the plasma membrane, necrosis results in release of intracellular constituents (i.e., alarmins, DAMPs) in the extracellular environment, which in turn activate innate immune cells [54]. This may represent a probable reason for IRI being correlated to a higher incidence of acute rejection after liver transplantation [55]. Apart from this, newly described alternative cell death pathways, predominantly necroptosis and ferroptosis, have gained considerable relevance in models of oxidative stress and tissue injury in recent years [9]. However, its precise role in the context of liver ischemia remains to be defined.

Nonetheless, the apoptotic fraction of hepatic IR injury still takes an even more important position in most of the current study models as potential proapoptotic enzyme pathways are relatively well measurable, thus creating a practicable approach towards cell death. However, former studies have not yet been able to fully show which exact pathways actually underlie the mediated cell death resulting in hepatic IRI. The consequent approach towards a scientific discrimination between the different types of cell death may be the elucidation of specific signaling cascades and thus defining the role of cell death under IR conditions.

Our data show that application of Baicalein, which acts via inhibition of 12/15-LOX besides its antioxidant function, significantly decreases cell death in a murine hepatic IRI model. Although our data does not prove any specific type of cell death model, we hypothesize that cell death in our experiments is primarily not caused by unregulated necrosis, as necrosis normally takes more than 3 hours to become apparent [53]. More likely, other types of cell death, such as necroptosis or the recently described mechanism of ferroptosis, may represent the underlying mechanism. The hypothesis of ferroptosis is supported by the results obtained by Xie et al. in 2016, in which beneficial effects of Baicalein on ferroptosis in pancreatic cancer could be demonstrated [56]. We thus speculate in line with previous findings in hepatic cells [57] that the GPX4-dependent death signaling, in which 12/15-LOX is still considered to represent one of the key enzymes leading towards cell death, does play a major role in hepatic IRI. Moreover, the central role of GPX4 in ferroptotic cell death has been thoroughly investigated in the past years [11, 58, 59]. Regarding these data and taking Seiler et al.'s and Loscalzo's results of 2008 into account, it might legitimately be hypothesized that sustained oxidative stress initiated by ischemia-reperfusion on the one hand leads to an impairment of GPX4 function and as a consequence stimulates the activation of 12/15-LOX increasing the so-called intracellular peroxide tone [23, 26]. Our data further suggest that the majority of hepatic IR injury is affected by more than just necrosis and alternative cell death pathways including apoptosis as also several proapoptotic enzymes were found to be significantly altered.

In 2008, AIF translocation was described as the main effector leading to cell death, whereas more recent studies have casted doubts about this hypothesis. 12S-HpETE, the product of activated 12/15-LOX, was pointed out as a possibly relevant mediator for caspase-independent cell death via AIF translocation, as no caspase-3 activation could be detected in GPX4-knockout cells [23, 26]. On the other hand, other studies have depicted caspase-3 as one of the key mediators in hepatic IRI [60, 61], so in vivo situation might be different. Our data show that the inhibiton of 12/15-LOX by Baicalein treatment of mice leads to a significant decrease of caspase-3, PARP, and JNK activation in liver tissue. We suggest that these players need to be considered as in a caspase-dependent setting as a part of the whole model of hepatic IRI. While we still consider ERK1/2 as a relevant mediator of hepatic IR injury, although it did not reach statistical significance, kinetic analyses of ERK have shown that the phosphorylation of ERK peaks within the first 20 minutes after the cell death stimulus, followed by a rather quick decay [62]. Our in vivo model thus indicated a tendency in negative regulation of ERK1/2 activation with the apoptotic stimulus being oxidative stress at the reperfusion phase. As this tendency was still measurable in a relevant manner even after 90 minutes post stimulus, we propose this pathway to be relevant in hepatic IRI. However, from our experience and regarding the most recent findings concerning the importance of GPX4 in ferroptosis, we consider that apoptosis is not the sole form of cell death under the conditions of hepatic warm ischemia. Other forms of regulated cell death, such as necroptosis and ferroptosis, thus have to be considered as well. Due to their previously described role in cell death development in IRI [61, 63], PARP and caspase-3 might hypothetically be relevant cell death mediators in the described pathways. JNK and ERK are known to be broad indicators of cell stress response and therefore located upstream [64, 65], so that the antioxidant influence of Baicalein might most probably explain their decrease after pretreatment. The exact correlation between certain mediators and respective forms of cell death that occur in hepatic IRI is still to be scrutinized in future experiments.

Glutathione hemostasis is known to have a direct influence on oxidative stress and its effects in IRI [34]. This study showed that an upregulation of glutathione biosynthesis is directly associated with a decreased amount of cell death, leading to decreased liver damage after hepatic IRI. As reviewed recently by Rodriguez-Lara et al., the relevance of oxidative stress in IRI was pointed out as the formation of ROS is one of the main events leading to organ damage [8]. We therefore state that the beneficial effects of 12/15-LOX inhibition observed in this study must primarily be considered a reduction of oxidative stress, potentially influenced by GPX4 enhancement and furtherly leading to lesser ROS formation.

DMSO was used as the carrier solution for Baicalein in this study. DMSO has already been described being hepatoprotective in several previous studies [66, 67]. Until now, the protective effects of DMSO in IRI have been imputed to decreased Kupffer cell activation and leukocyte adhesion [68], as well as restoration of vitamin C levels attenuating oxidative stress [69]. The important role of Kupffer cells in hepatic IRI [70] and their association with reactive oxygen species [12, 71] have already been shown. Our data suggest that DMSO might also have a direct effect on hepatic IRI by decreasing the number of dying cells and slightly enhancing glutathione oxidation itself. We could show that DMSO application led to a reduced loss of cell viability on one hand. On the other hand, it decreased the activation levels of PARP and JNK after hepatic ischemia and reperfusion, yet in a more discrete manner than Baicalein. Nonetheless, Baicalein treatment resulted in significantly lowered cell death, potentially due to its effects on caspase-3 and ERK1/2 which were not affected by DMSO pretreatment. We thus hypothesize that DMSO might interfere with the described death pathway, yet not in the same and efficient way as inhibition of 12/15-LOX. This might be—in contrast to Baicalein pretreatment—due to the lack of alteration in caspase-3 activity, one of the hepatic IRI's key enzymes [60, 61].

In general, our data support the models of antagonistic interaction between 12/15-LOX and GPX4 that have been described previously [23, 26]. As Baicalein is known to be an effective inhibitor of 12/15-LOX [40], we could show that Baicalein administration indeed leads to significant protection against postischemic hepatic cell death by reduction of oxidative stress. This results in increased glutathione oxidation, as measured by a relatively bigger amount of its oxidized product GSSG versus the reduced substrate GSH. As GPX4 has been described to directly decrease the peroxide tone within the cell by reducing ROS which are potent activators of 12/15-LOX [23], it may be speculated that Baicalein not only decreases the peroxide tone by its antioxidant effects but also by increasing glutathione metabolism, hence presumably GPX4 activity. Both effects cause a decreased 12/15-LOX activation. The exact mechanisms of cell death induction by 12/15-LOX still have to be precisely elucidated (i.e., by means of KO individuals within the experiments) also in light of the fact that hepatocyte-specific deletion of GPX4 causes massive cell death of hepatocytes and perinatal lethality of mice [57].

Having pointed out the protective effects following Baicalein and DMSO administration, we could also demonstrate that the administered agents lead to a significant increase of transaminases and glutamate dehydrogenase in sham-operated mice. We interpret this adverse effect induced by DMSO and Baicalein themselves. Although this could point to conflicting results as opposed to the protective effects mentioned above, an increase of TUNEL positivity could not be monitored. Therefore, we conclude that the administered agents provoke some cell damage without affecting liver synthesis, biliary excretion, or renal function resulting in a release of hepatic enzymes without affecting cell structures relevant in inducing cell death. Several previous studies regarding the potential toxicity of DMSO support our observations of transaminase elevation [72], especially when taking into account that we used a high dosage of DMSO (6600 mg/kg body weight) in order to allow dosing of high concentrations of Baicalein (120 mg/kg body weight i.p.) at maximum solubility in DMSO in this proof-of-principle experiment. The applied dosage of Baicalein is well established as it has been used in previously described animal studies [73, 74], and preliminary dosage finding experiments of our study group showed no significant impact at lower concentrations. Previous studies regarding the protective effects of DMSO on hepatic ischemia and reperfusion used significantly lower dosages (500 mg/kg b.w.), which did not lead to elevated AST, ALT, and GLDH levels [66, 67]. Our observations relating to toxicity of DMSO and Baicalein were substantiated as a brief decrease in blood pressure with a concomitant increase of heart rate could be monitored after drug administration. Although quickly self-limiting, one could describe this effect as drug toxicity of DMSO with or without Baicalein. Baicalein has been described to affect mitochondrial bioenergetics at high concentration and long exposure [75], as well as to increase toxic effects of other drugs like cisplatin by enhancing gap junction communications [76]. However, the concentration and exposure time in the present experiment was by far lower. Even though these results provide an indication of Baicalein's adverse effects, a thorough and sufficient insight in the toxicity of Baicalein is yet lacking.

Our hypothesis is supported by the observation that this increase of laboratory parameters measured in blood samples after DMSO or Baicalein could also be demonstrated in the IRI-induced mice groups. However, the increase of transaminase and GLDH, calculated as the relative increase in enzyme activation, was up to four times lower than between the sham-operated groups after Baicalein treatment. This facilitates the hypothesis that the significantly reduced increase of liver enzymes might be due to the cell protective effects as mentioned before. Concordantly to our previously described results, this became more evident after Baicalein pretreatment than after sole DMSO application, predominantly in AST and GLDH levels. However, whether the toxic effects of a therapy targeting 12/15-LOX qualifies its protective value in long term concerning hepatic IRI is yet to be investigated.

Conclusively, it can be stated that the inhibition of 12/15-LOX prevents cell death caused by hepatic IRI by sustaining glutathione hemostasis—presumably involving GPX4—therefore attenuating oxidative stress. Furthermore, our results suggest that the 12/15-LOX-dependent signaling cascade might relevantly impact on the key enzymes JNK, caspase-3, PARP, and ERK and thus plays a major role within the development of hepatic IRI. After further scientific evaluation, this could be considered as a starting point for developing therapeutic approaches to minimize hepatic IRI in major liver surgery and OLT, as long as a reasonable relation between toxicity and protectiveness can be achieved.

## Figures and Tables

**Table 1:** Serum analyses.

	Sham			IRI		
	Control	DMSO	Baicalein	Control	DMSO	Baicalein
Creatinine (mg/dl)	0.8 ± 0	0.8 ± 0	0.8 ± 0	0.8 ± 0.0001	0.8 ± 0.0001	0.8 ± 0.0001
Bilirubin (mg/dl)	0.9 ± 0.1	1.1 ± 0.3	1.0 ± 0.2	1.1 ± 0.2	1.4 ± 0.3	1.3 ± 0.1
Cholinesterase (kU/l)	2.9 ± 0.1	3.2 ± 0.2	3.5 ± 0.2	2.9 ± 0.1	3.3 ± 0.1	3.3 ± 0.2
AST (U/l)	152.0 ± 34.3	315.8 ± 65.4	627.0 ± 102.0	697.2 ± 180.7	1162.8 ± 310.5	1780.5 ± 455.7
ALT (U/l)	121.0 ± 26.5	269.0 ± 73.781	243.0 ± 78.1	860.0 ± 291.7	1280.8 ± 427.0	1593.1 ± 626.9
GLDH (U/l)	14.5 ± 3.8	39.5 ± 18.3	89.8 ± 21.1	131.3 ± 25.7	230.3 ± 68.3	363.8 ± 132.9

Data presented as mean ± SEM. No significant differences between all groups could be stated regarding creatinine, bilirubin, and cholinesterase values. A relevant elevation of hepatic damage enzymes could be observed after Baicalein and sole DMSO application compared to the respective control in both Sham and IRI groups.
